# Grouped Feature Representation and Gated Multilayer Perceptron for Event-Level Football Pass Outcome Prediction

**DOI:** 10.3390/e28060703

**Published:** 2026-06-17

**Authors:** Yijuan Yuan, Shaosong Wang, Yonghong Deng, Zhibin Li

**Affiliations:** 1 Department of Physical Education, Liaocheng University Dongchang College, Liaocheng 252000, China; 1120111@lcudcc.edu.cn; 2 Department of Basic Courses, Liaocheng Vocational and Technical College, Liaocheng 252000, China; 3 Sichuan Provincial Promotion Center of Digital Transformation, Chengdu Technological University, Chengdu 611730, China; 4 School of Software Engineering, Chengdu University of Information Technology, Chengdu 610225, China; lizhibin@cuit.edu.cn

**Keywords:** football pass outcome prediction, grouped feature representation, event-level modeling, student football, physical education

## Abstract

Accurate prediction of football pass outcomes is important for tactical analysis, decision evaluation, and skill-oriented feedback in student football training and physical education. However, event-level pass outcome prediction remains challenging because pass success is jointly influenced by spatial context, defensive pressure, receiver-related cues, and historical coordination between players. To address this issue, this study proposes an information-guided multilayer perceptron (IGMLP) based on grouped feature representation and gated feature fusion using structured event data. In the proposed framework, input variables are organized into interpretable semantic feature groups, including contextual features, pressure-aware features, historical coordination features, and receiver-related features. These groups are encoded through separate branches and adaptively fused by a group-level gating mechanism for nonlinear pass outcome modeling. Unlike conventional gated neural architectures that usually apply generic gates to hidden units, channels, or sequential states, the proposed gated design operates at the semantic feature-group level and adaptively weights football-specific information sources according to their relevance to each pass event. Using the StatsBomb open-event dataset, both prediction and recognition paths were constructed, and the proposed model was compared with standard multilayer perceptron (MLP), residual neural network (ResNet), boosting tree (BT), convolutional neural network (CNN), and long short-term memory network (LSTM). In the prediction path, IGMLP achieved an Accuracy of 0.9184, Precision of 0.9295, Recall of 0.9837, F1-score of 0.9558, and AUC of 0.9325. In the recognition path, IGMLP achieved an Accuracy of 0.9808, Precision of 0.9882, Recall of 0.9902, F1-score of 0.9893, and AUC of 0.9925. These results indicate that semantic feature grouping and gated feature fusion are effective for event-level football pass outcome prediction.

## 1. Introduction

In modern football analytics, a match is increasingly viewed not only as a final score or team-level result, but also as a sequence of fine-grained events that jointly shape possession, territorial progression, and attacking opportunities [1]. Passing is one of the most frequent and tactically meaningful event types in this sequence, because it directly reflects how players exchange information, respond to defensive pressure, and construct collective attacking patterns. Predicting whether an individual pass will be completed is therefore an important event-level task for understanding decision quality, player cooperation, and team tactical execution [2]. Unlike match outcome prediction, which usually relies on aggregated indicators such as team strength, historical results, or player statistics, pass outcome prediction requires a more detailed description of the local game context now of action [3,4]. The success of a pass may depend on spatial variables such as pass distance, angle, and field zone, as well as contextual factors such as defensive pressure, receiver availability, possession history, and the interaction between the passer and potential teammates. These factors are often coupled in nonlinear ways, making it difficult for simple statistical models to capture the underlying decision patterns. Consequently, there is a need to develop a data-driven prediction framework that can integrate informative event-level features and identify the key cues associated with successful or unsuccessful football passes.

Currently, a growing body of research has investigated football analytics and prediction from different perspectives, including match outcome forecasting, event-level modeling, tactical behavior recognition, player performance assessment, and sports-related decision support. In match-level prediction, Sun and Chu [5] proposed a deep learning-based quantum neural network for football match outcome prediction and reported improved predictive performance on historical European league data. Quan and Luo [6] further developed a CNN-BiLSTM-Att model by integrating player compatibility and dynamic lineup information, showing the potential of deep learning architectures in football match-winning probability prediction. To improve computational efficiency, Li and Hong [7] introduced an edge computing and machine learning-based framework for football match result prediction by deploying feature extraction and classification tasks on edge nodes. From a statistical modeling perspective, Lee et al. [8] proposed a Bayesian hierarchical Poisson model to quantify changes in home advantage during spectator-free football matches after the COVID-19 pandemic, while Stock et al. [9] extended a stochastic football league model by incorporating team potential and home advantage within Monte Carlo simulations. These studies demonstrate that statistical learning, deep learning, and distributed computing methods have been widely applied to football match prediction; however, most of them focus on team-level or league-level outcomes rather than fine-grained in-game actions.

With the increasing availability of event and tracking data, researchers have begun to explore more detailed football behaviors and tactical patterns. Alves [10] proposed a convolutional online prediction approach to forecast the next event in a football match using historical event information from elite European leagues, indicating that event sequences contain valuable information for real-time football analysis. Xie [11] developed a CNN-based framework for multi-target motion prediction and tracking management in football matches to improve the real-time accuracy of tactical image analysis. Kausalya and Sudha [12] combined YOLOv5, MixNetCNN, and the Mayfly optimization algorithm for football player detection and action classification, achieving improved performance in vision-based action recognition. Although these studies are closer to in-game behavior modeling, they mainly focus on next-event forecasting, visual tracking, or action classification, while the prediction of whether a specific pass can be successfully completed remains insufficiently explored.

In addition, artificial intelligence and statistical models have been applied to other football-related prediction tasks. Sun and Gu [13] developed optimized Decision Tree Regression and Random Forest Regression models for football player market value prediction, providing support for transfer market decision-making. Pan [14] proposed an improved LSTM-based W-LSTM model for football players’ physical training optimization and prediction, while Liao and Fu [15] constructed a CNN-based key point detection model for youth football training. Dijkhuis et al. [16] used elite soccer tracking data to predict individual players’ physical performance in the early phase of a match, showing that Random Forest models can support substitution-related decisions. Beyond performance and training analysis, Hassan et al. [17] applied a Multi-Head Transformer-based neural network to cybersecurity incident severity prediction in professional football organizations, and Empacher et al. [18] developed statistical point and interval prediction methods for sports analytics. These studies broaden the application scope of intelligent modeling in football-related scenarios, but they do not directly address event-level pass outcome prediction.

More directly related to the present task, pass evaluation has been investigated in soccer analytics through pass completion probability, pass difficulty estimation, risk–reward assessment, and action-value frameworks. Anzer et al. [19] proposed an expected passes model to estimate pass completion probability and pass difficulty using spatio-temporal data. Power et al. [20] objectively measured the risk and reward of passes in soccer from tracking data, and Goes et al. [21] further developed a risk–reward assessment framework for passing decisions across different positional roles. In addition to direct pass evaluation, several possession-value and action-value models have been widely used to assess the tactical contribution of passes and other on-ball actions. Hassani et al. [22] introduced the expected threat model to evaluate how ball movement changes attacking threat, while Fernández et al. [23] proposed a pitch-control-based framework for measuring space creation in professional soccer. Fernández et al. [24] further developed an expected possession value framework for fine-grained evaluation of soccer possessions and potential actions. Similarly, Decroos et al. [25] proposed the VAEP framework to value player actions by estimating how each action changes the probabilities of scoring and conceding in subsequent game states. These studies provide important foundations for pass evaluation and modern soccer analytics.

Taken together, existing studies have made important progress in football match prediction, tactical analysis, player evaluation, pass evaluation, possession-value modeling, and other football-related decision tasks. Nevertheless, the objective of the present study is different from most existing pass evaluation and possession-value frameworks. Previous studies mainly focus on pass difficulty estimation, action-value assessment, player contribution evaluation, or risk–reward analysis, and some of them rely on tracking data, pitch-control information, or proprietary annotations. In contrast, this study focuses on lightweight event-level pass outcome prediction using structured open-event data. The key objective is to examine whether football-specific semantic feature groups, including contextual, pressure-aware, historical coordination, and receiver-related information, can be adaptively fused by a gated MLP model to improve pass success prediction.

**Figure 1:**
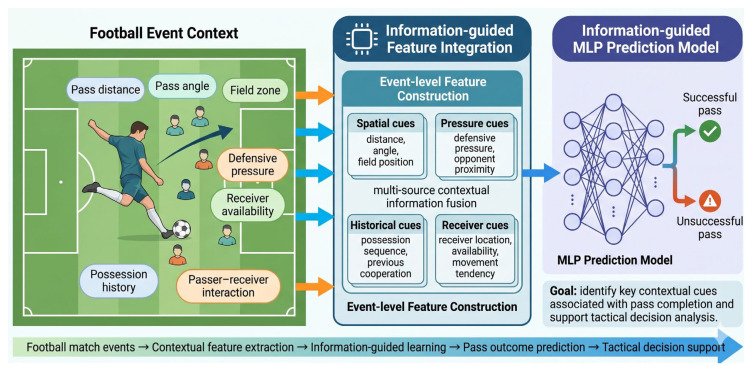
Overall framework of event-level football pass outcome prediction based on information-guided learning.

As illustrated in Figure 1, the proposed framework starts from fine-grained football match events and extracts multiple contextual cues related to a specific passing action, including spatial cues, pressure cues, historical cues, and receiver-related cues. These heterogeneous features are then integrated into an information-guided representation and fed into the MLP prediction model to determine whether the pass is likely to be completed successfully. In this way, the proposed framework links local in-game context with pass completion outcomes and provides a data-driven basis for tactical decision analysis.

The contributions of this study can be summarized as follows:

(a) A grouped feature representation framework is proposed for event-level football pass outcome prediction. This framework organizes passing information from multiple perspectives, including local game context, defensive pressure, historical cooperation, and receiver-related cues, which enables a more detailed characterization of the passing decision environment.
(b) A gated multilayer perceptron model with semantic group-level fusion is developed to predict pass outcomes from structured event-level data. The model captures nonlinear relationships among informative passing features and provides an efficient and practical prediction framework for football performance analysis and sports education.

The remainder of this paper is organized as follows. Section 2 describes the dataset and problem formulation. Section 3 introduces the proposed IGMLP model. Section 4 presents the experimental results and discussion. Section 5 concludes this study and discusses future work.

## 2. Dataset and Problem Formulation

### 2.1. Data Source and Sample Construction

The dataset used in this study was derived from the publicly available StatsBomb open-event data [26]. Specifically, this study used La Liga matches from six seasons, namely 2015/2016, 2016/2017, 2017/2018, 2018/2019, 2019/2020, and 2020/2021. After event extraction and sample cleaning, the dataset covered 231 matches. The StatsBomb event data provide structured event-level annotations, including event type, player identity, team information, match context, spatial coordinates, event timestamps, pass attributes, pressure information, and related ball-receipt information. These annotations provide a suitable foundation for football pass outcome prediction because they describe both the geometric properties of passes and the surrounding tactical context.

To construct the modeling dataset, the original event streams were first re-extracted into a modeling-ready table containing 100,000 candidate pass samples and 65 variables. Only open-play pass events were retained, while non-open-play situations, such as set pieces, corners, free kicks, throw-ins, kick-offs, and other restart-related events, were excluded because their tactical contexts differ from regular open-play passing situations. The data were then cleaned by removing samples with missing pass outcome labels, missing pass start or end locations, missing pass length, missing pass angle, or missing intended receiver information. After preprocessing and feature construction, the final dataset contained 95,747 valid open-play pass samples and 88 variables.

During preprocessing, samples with missing pass outcome labels were first removed. Samples with missing essential geometric variables, including pass start location, pass end location, pass length, and pass angle, were also excluded because these variables are necessary for constructing pass-level spatial features. In addition, when the intended receiver identifier was available, samples without valid receiver information were removed. For non-essential derived numerical variables, missing values were replaced with zero after feature construction. For receipt-related variables, missing receipt time gaps and location errors were assigned a large placeholder value to indicate unmatched or unavailable receipt information.

Each sample corresponds to one event-level pass instance. The target label was defined according to whether the pass was successfully completed. A pass was labeled as successful if the intended receiving side retained possession through the corresponding passing event, and failed otherwise. Among the final 95,747 samples, 85,353 were successful passes and 10,394 were unsuccessful passes, corresponding to an overall success rate of 89.14%.

To reduce information leakage from future matches into earlier observations, all samples were sorted chronologically according to match date and kick-off time before dataset partitioning. The dataset was then divided into training, validation, and test subsets using a 70%/15%/15% split. The training set contained 67,022 samples, the validation set contained 14,362 samples, and the test set contained 14,363 samples. The training set was used for model parameter optimization, the validation set was used for threshold selection and model monitoring, and the test set was used only for final performance evaluation. The detailed label distribution is reported in Table 1.

To improve interpretability and better match the structure of football decision-making, the input variables were organized into several semantically meaningful feature groups. In the main prediction path, three feature groups were used: contextual features, pressure-aware features, and historical coordination features. Contextual features describe the spatial and match-state conditions of a pass, including pass location, pass length, pass angle, forward progression, distance-to-goal-related variables, match period, possession index, score state, play pattern, pass height, pass type, and passer position. Pressure-aware features describe the local difficulty of a pass under defensive disturbance, including the under-pressure indicator, pressure-related interaction variables, and special pass-type indicators such as switch passes, crosses, and cut-backs. Historical coordination features describe the previous cooperative relationship between the passer and the intended receiver, including historical total passes, successful and failed historical connections, pairwise success rate, shared match information, trust score, risk score, creativity score, and pressure-history interaction variables. For the auxiliary recognition path, receipt-related post-event features were additionally included. These variables include whether a corresponding receipt event was found, the receipt location, the time gap between the pass and receipt events, the location error between the intended endpoint and actual receipt location, and the receiver position. Because these receipt-related variables become available only after the pass has unfolded, they were used only for auxiliary recognition analysis and were not included in the main prediction path.

For model input construction, numerical variables were standardized using the mean and standard deviation estimated from the training set. The same normalization parameters were then applied to the validation and test sets. Categorical variables were converted into one-hot representations based on the categories observed in the training data. After one-hot encoding, the input dimensions of the prediction path were 55 for contextual features, 6 for pressure-aware features, and 15 for historical coordination features. In the recognition path, the receipt-related branch had an input dimension of 30.

**Figure 2:**
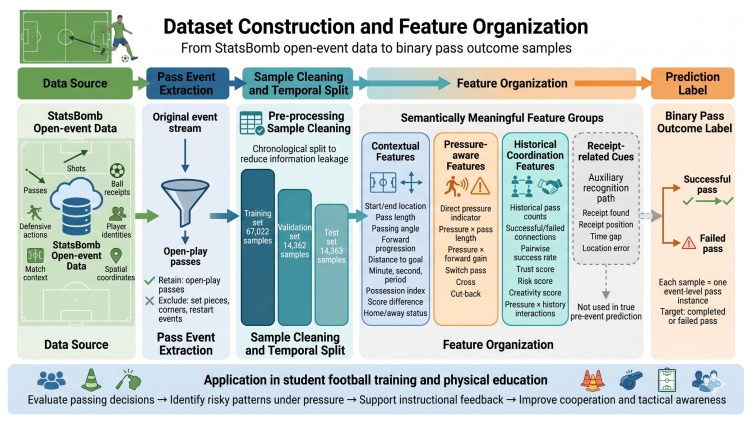
Dataset construction and feature organization for event-level football pass outcome prediction.

Figure 2 summarizes the dataset construction and feature organization procedure used in this study. Starting from the StatsBomb open-event data, open-play pass events were extracted and cleaned, and the resulting samples were chronologically divided into training, validation, and test sets to reduce potential information leakage. The input variables were then organized into contextual, pressure-aware, and historical coordination feature groups for the main prediction path, while receipt-related variables were used only for the auxiliary recognition path. This workflow provides a clear data foundation for the subsequent IGMLP modeling framework.

### 2.2. Problem Formulation

To define the event-level input representation, the historical coordination variables were specified before the classification problem was formulated. For the *i*-th pass, let $N_{i}$ denote the historical total number of passes between the same passer–receiver pair before the current event, $S_{i}$ denote the historical number of successful passes, and $F_{i}$ denote the historical number of failed passes. The pair trust score was defined as the smoothed historical success ratio:

(1) $$T_{i}=\frac{S_{i}}{N_{i}+1}$$

The pair risk score was defined as the smoothed historical failure ratio:

(2) $$R_{i}=\frac{F_{i}}{N_{i}+1}$$

The pair creativity score was defined according to whether the passing relationship was associated with shot-assist or goal-assist events:

(3) $$C_{i}=\frac{A_{i,shot}+A_{i,goal}}{N_{i}+1}$$

where $A_{i,shot}$ and $A_{i,goal}$ denote the shot-assist and goal-assist indicators, respectively. In addition, pressure-history interaction features were constructed by multiplying the current under-pressure indicator with historical coordination variables, including successful historical connections, failed historical connections, and pair trust score. These definitions provide the basis for representing both accumulated passer–receiver cooperation and its interaction with current defensive pressure.

In this study, football pass outcome modeling is formulated as an event-level binary classification problem, where the objective is to determine whether a given pass will be completed successfully under the available event information. Let the *i*-th pass event be represented by a grouped input feature vector, and let its corresponding output label indicate the pass outcome. The prediction task can be expressed as follows:

(4) $$x_{i}=[x_{i}^{\left(c\right)},x_{i}^{\left(p\right)},x_{i}^{\left(h\right)}]$$

where $x_{i}$ denotes the overall input representation of the *i*-th pass sample, $x_{i}^{\left(c\right)}$ denotes the contextual feature group, $x_{i}^{\left(p\right)}$ denotes the pressure-aware feature group, and $x_{i}^{\left(h\right)}$ denotes the historical coordination feature group.

More specifically,

(5) $$x_{i}^{\left(c\right)}\in {\mathbb{R}}^{d_{c}},\;x_{i}^{\left(p\right)}\in {\mathbb{R}}^{d_{p}},\;x_{i}^{\left(h\right)}\in {\mathbb{R}}^{d_{h}}$$

where $d_{c}$, $d_{p}$, and $d_{h}$ are the dimensionalities of the contextual, pressure-aware, and historical coordination feature groups, respectively. Accordingly, the total input dimension is given by

(6) $$d=d_{c}+d_{p}+d_{h}$$

here, *d* represents the dimension of the complete input feature vector $x_{i}$.

For the auxiliary recognition path, receipt-related post-event cues can be further incorporated. In that case, the input representation becomes

(7) $$x_{i}^{\left(r\right)}=[x_{i}^{\left(c\right)},x_{i}^{\left(p\right)},x_{i}^{\left(h\right)},x_{i}^{\left(q\right)}]$$

where $x_{i}^{\left(r\right)}$ denotes the extended input representation in the recognition path, and $x_{i}^{\left(q\right)}\in {\mathbb{R}}^{d_{q}}$ denotes the receipt-related feature group, and $d_{q}$ is its corresponding dimension. Since $x_{i}^{\left(q\right)}$ becomes available only after the pass event unfolds, it is not included in the main prediction path and is used only for auxiliary recognition analysis.

For each pass event, the output label is defined as

(8) $$y_{i}\in \{0,1\}$$

where $y_{i}=1$ indicates that the *i*-th pass is successful, and $y_{i}=0$ indicates that the pass is unsuccessful.

Accordingly, the objective of the model is to learn a mapping function from the grouped input features to the pass outcome label:

(9) $${\hat{y}}_{i}=f(x_{i};\Theta )$$

where *f*(⋅) denotes the prediction model, Θ denotes the set of trainable model parameters, and ${\hat{y}}_{i}$ denotes the predicted output for the *i*-th sample.

Since the task is formulated as binary classification, the model estimates the probability that a pass is successful:

(10) $${\hat{p}}_{i}=P(y_{i}=1|x_{i};\Theta )$$

where ${\hat{p}}_{i}$ denotes the predicted probability that the *i*-th pass will be completed successfully. Based on this probability, the final class prediction is obtained by thresholding:

(11) $${\hat{y}}_{i}=\left\{\begin{array}{l} 1,{\hat{p}}_{i}\geq \tau \\ 0,{\hat{p}}_{i}\leq \tau \end{array}\right.$$

where *τ* ∈ (0,1) denotes the decision threshold. In this study, *τ* is selected on the validation set to achieve better classification balance.

Given a dataset containing *N* pass samples,

(12) $$D={\left\{(x_{i},y_{i})\right\}}_{i=1}^{N}$$

where D denotes the full event-level pass dataset, *N* denotes the total number of samples.

To this end, the binary cross-entropy loss is adopted:

(13) $$L(\Theta )=-\frac{1}{N}\sum_{i=1}^{N}\left[y_{i}\log({\hat{p}}_{i})+(1-y_{i})\log(1-{\hat{p}}_{i})\right]$$

where L(Θ) denotes the loss function used for model training.

Therefore, the main prediction problem considered in this study can be summarized as

(14) $${\hat{y}}_{i}=f(x_{i}^{\left(c\right)},x_{i}^{\left(p\right)},x_{i}^{\left(h\right)};\Theta )$$

while the auxiliary recognition problem can be written as

(15) $${\hat{y}}_{i}=f(x_{i}^{\left(c\right)},x_{i}^{\left(p\right)},x_{i}^{\left(h\right)},x_{i}^{\left(q\right)};\Theta )$$

From the application perspective, the prediction path is the core setting of this study because it uses only pre-event or event-synchronous information and therefore better reflects the realistic task of evaluating pass decisions before the outcome is fully observed. This setting is particularly meaningful for student football training and physical education, where instructors are more concerned with passing decision quality, tactical awareness, and coordination ability. By contrast, the recognition path includes additional post-event cues and is therefore treated only as an auxiliary analysis setting rather than the main prediction task.

## 3. Proposed Information-Guided Multilayer Perceptron

### 3.1. Overall Framework

To effectively exploit heterogeneous event information in football pass outcome prediction, this study proposes an IGMLP. Unlike a standard multilayer perceptron that directly concatenates all features into a single input vector, IGMLP explicitly preserves the grouped structure of football event information and processes different feature groups through separate encoding branches before adaptive fusion.

For the main prediction path, the grouped input of the *i*-th pass sample is given by Equation (4), namely $x_{i}=[x_{i}^{\left(c\right)},x_{i}^{\left(p\right)},x_{i}^{\left(h\right)}]$. Each feature group is first encoded into a latent representation through its own branch. The resulting group-level representations are then aggregated through an information-guided gating mechanism, and the fused representation is finally mapped to the predicted probability ${\hat{p}}_{i}$.

Accordingly, the overall computation of IGMLP can be written as

(16) $$z_{i}^{\left(c\right)}=\phi_{c}(x_{i}^{\left(c\right)}),\;z_{i}^{\left(p\right)}=\phi_{p}(x_{i}^{\left(p\right)}),\;z_{i}^{\left(h\right)}=\phi_{h}(x_{i}^{\left(h\right)})$$

where $z_{i}^{\left(c\right)}\in {\mathbb{R}}^{m}$, $z_{i}^{\left(p\right)}\in {\mathbb{R}}^{m}$, and $z_{i}^{\left(h\right)}\in {\mathbb{R}}^{m}$ denote the latent representations of the contextual, pressure-aware, and historical coordination feature groups, respectively; m is the latent embedding dimension; and $\phi_{c}(\cdot )$, $\phi_{p}(\cdot )$ and $\phi_{h}(\cdot )$ denote the branch encoders.

The three branch representations are then fused by a gated weighting mechanism:

(17) $$z_{i}=\sum_{u\in \{c,p,h\}}g_{i}^{\left(u\right)}z_{i}^{\left(u\right)}$$

where $z_{i}\in {\mathbb{R}}^{m}$ is the fused representation of the *i*-th pass sample, and $g_{i}^{\left(u\right)}$ denotes the gate weight assigned to the *u*-th feature group.

The final probability output is obtained by

(18) $${\hat{p}}_{i}=\sigma (\psi (z_{i}))$$

where *ψ*(⋅) denotes the output mapping of the classifier head and *σ*(⋅) denotes the sigmoid activation function.

Therefore, IGMLP transforms the grouped football event input into a compact discriminative representation by combining group-wise feature encoding and adaptive information-guided fusion. This design is particularly suitable for event-level football pass prediction because contextual, pressure-related, and historical coordination information have distinct semantic meanings and should not be indiscriminately mixed at the input stage.

**Figure 3:**
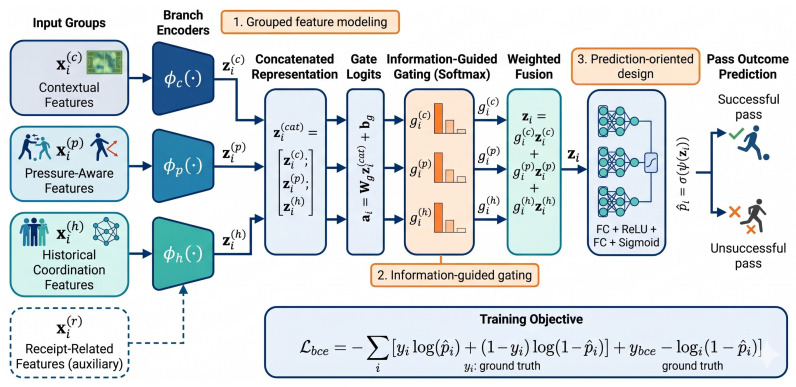
Overall architecture of the proposed IGMLP for event-level football pass outcome prediction.

Figure 3 illustrates the overall architecture of the proposed IGMLP framework. The model takes grouped event-level features as input, performs branch-wise feature encoding for contextual, pressure-aware, and historical coordination information, and then adaptively fuses these representations through an information-guided gating mechanism for pass outcome prediction.

As shown in Figure 3, the proposed framework consists of three main stages: group-wise feature encoding, information-guided gated fusion, and classification-based probability estimation. The prediction path uses only pre-event and event-synchronous information, while the receipt-related branch is included only in the auxiliary recognition path. The detailed formulations of these components are presented in the following subsections.

### 3.2. Group-Wise Feature Encoding

The first key component of IGMLP is the group-wise encoding strategy. For each feature group, a dedicated multilayer perceptron branch is used to project the original input into a latent space of dimension m. This allows each branch to learn a group-specific nonlinear transformation.

For a generic feature group *u* ∈ {*c*, *p*, *h*}, the branch encoder is defined as

(19) $$h_{i}^{\left(u\right)}=\rho (W_{1}^{\left(u\right)}x_{i}^{\left(u\right)}+b_{1}^{\left(u\right)})$$

(20) $$z_{i}^{\left(u\right)}=\rho (W_{2}^{\left(u\right)}h_{i}^{\left(u\right)}+b_{2}^{\left(u\right)})$$

where $W_{1}^{\left(u\right)}$, $W_{2}^{\left(u\right)}$ are trainable weight matrices, $b_{1}^{\left(u\right)}$, $b_{2}^{\left(u\right)}$ are trainable bias vectors, $h_{i}^{\left(u\right)}$ denotes the hidden representation of the *u*-th branch, and *ρ*(⋅) denotes the nonlinear activation function.

According to Equations (16) and (17), each information group is independently encoded before fusion. This has two advantages. First, it preserves the semantic heterogeneity of football event information. Second, it prevents different feature groups from interfering with each other too early in the learning process.

For the auxiliary recognition path, the same encoding principle is extended to the receipt-related group:

(21) $$z_{i}^{\left(q\right)}=\phi_{q}(x_{i}^{\left(q\right)})$$

where $\phi_{q}(\cdot )$ denotes the encoder of the receipt-related branch. Thus, the recognition path contains four branch representations, while the prediction path contains three.

### 3.3. Information-Guided Gating Mechanism

The second key component of IGMLP is the information-guided gating mechanism. Instead of directly concatenating $z_{i}^{\left(c\right)}$, $z_{i}^{\left(p\right)}$ and $z_{i}^{\left(h\right)}$, the model first evaluates their relative importance and then assigns adaptive gate weights to different information groups.

The branch representations are first concatenated as

(22) $$z_{i}^{\left(cat\right)}=[z_{i}^{\left(c\right)};z_{i}^{\left(p\right)};z_{i}^{\left(h\right)}]$$

where $z_{i}^{\left(cat\right)}\in {\mathbb{R}}^{3m}$ denotes the concatenated latent representation in the prediction path. Based on $z_{i}^{\left(cat\right)}$, the gate logits are computed by

(23) $$a_{i}=W_{g}z_{i}^{\left(cat\right)}+b_{g}$$

where $a_{i}\in {\mathbb{R}}^{3}$ denotes the gate-logit vector, $W_{g}$ is the gate weight matrix, and $b_{g}$ is the gate bias vector.

The gate weights are then normalized through the softmax function:

(24) $$g_{i}^{\left(u\right)}=\frac{\exp(a_{i}^{\left(u\right)})}{\sum_{u\in \{c,p,h\}}\exp(a_{i}^{\left(u\right)})},u\in \{c,p,h\}$$

where $a_{i}^{\left(u\right)}$ denotes the *u*-th element of $a_{i}$. From Equation (24), the gate weights satisfy

(25) $$\sum_{u\in \{c,p,h\}}g_{i}^{\left(u\right)}=1,\;g_{i}^{\left(u\right)}\geq 0$$

Therefore, the fused representation in Equation (17) can be regarded as a convex combination of the branch-level latent representations. This means that the model automatically allocates more emphasis to the information group that is more useful for the current pass sample.

The use of SoftMax gating is motivated by its normalized group-level weighting property. Unlike independent sigmoid gates, which may assign high weights to multiple feature groups simultaneously without normalization, SoftMax gating constrains the gate weights to be comparable and to compete. Therefore, the resulting weights can indicate the relative contribution of contextual, pressure-aware, and historical coordination information for each pass sample. This is consistent with the objective of the proposed model, because the success of a pass may depend on different information sources under different game contexts. For example, some passes may be mainly determined by spatial context, whereas others may be more strongly affected by defensive pressure or previous passer–receiver coordination. Thus, the gate in IGMLP is used as a semantic group-level fusion mechanism rather than a generic hidden-unit gate.

Substituting Equation (24) into Equation (17), the fused representation can be rewritten as

(26) $$z_{i}=\sum_{u\in \{c,p,h\}}g_{i}^{\left(u\right)}z_{i}^{\left(u\right)}=\frac{\exp(a_{i}^{\left(c\right)})z_{i}^{\left(c\right)}+\exp(a_{i}^{\left(p\right)})z_{i}^{\left(p\right)}+\exp(a_{i}^{\left(h\right)})z_{i}^{\left(h\right)}}{\exp(a_{i}^{\left(c\right)})+\exp(a_{i}^{\left(p\right)})+\exp(a_{i}^{\left(h\right)})}$$

Equation (23) explicitly shows that IGMLP does not treat contextual, pressure-aware, and historical coordination information as equally important for every pass sample. Instead, the contribution of each group is adaptively determined by the learned gate values.

For the recognition path, the same mechanism is naturally extended by replacing Equation (19) with

(27) $$z_{i}^{\left(cat,r\right)}=[z_{i}^{\left(c\right)};z_{i}^{\left(p\right)};z_{i}^{\left(h\right)};z_{i}^{\left(q\right)}]$$

The corresponding gate normalization is performed over the index set {*c*, *p*, *h*, *q*}.

After information-guided fusion, the fused representation $z_{i}$ is passed through the classification head to estimate the success probability of the *i*-th pass event. Specifically, the classifier head is defined as

(28) $$\left\{\begin{array}{l} o_{i}=\rho (W_{o}z_{i}+b_{o})\\ s_{i}=W_{s}o_{i}+b_{s}\\ {\hat{p}}_{i}=\sigma (s_{i}) \end{array}\right.$$

where $o_{i}$ denotes the hidden output of the classifier head, $s_{i}$ denotes the scalar logit, $W_{o}$, $b_{o}$, $W_{s}$ and $b_{s}$ are trainable parameters, and ${\hat{p}}_{i}$ is the predicted success probability defined consistently with Equation (10).

The predicted class label is then obtained according to Equation (11). During training, the model parameters are optimized by minimizing the binary cross-entropy loss in Equation (13). By substituting Equation (27) into Equation (10), the optimization objective of IGMLP can be written as

(29) $$L(\Theta )=-\frac{1}{N}\sum_{i=1}^{N}\left[y_{i}\log(\sigma (s_{i}))+(1-y_{i})\log(1-\sigma (s_{i}))\right]$$

The complete parameter set of IGMLP is therefore given by

(30) $$\Theta ={\left\{W_{1}^{\left(u\right)},b_{1}^{\left(u\right)},W_{2}^{\left(u\right)},b_{2}^{\left(u\right)}\right\}}_{u}\cup \{W_{g},b_{g},W_{o},b_{o},W_{s},b_{s}\}$$

where the index set *u* is {*c*, *p*, *h*} for the prediction path and {*c*, *p*, *h*, *q*} for the recognition path.

Combining Equations (13)–(27), the prediction path of IGMLP can be summarized as

(31) $${\hat{p}}_{i}=\sigma (\psi (\sum_{u\in (c,p,h)}g_{i}^{\left(u\right)}\phi_{u}(x_{i}^{\left(u\right)})))$$

which explicitly describes the full process from grouped input encoding to adaptive information fusion and final probability estimation.

Algorithm 1 summarizes the training procedure of the proposed IGMLP. The main steps are consistent with the preceding mathematical formulation, including branch-wise encoding, information-guided gating, weighted fusion, and probability estimation. The binary cross-entropy loss in the algorithm has the same form as Equation (26), but it is computed over the current mini-batch B during stochastic training. Thus, *N* is replaced by |B|, and the summation is performed over the samples in B. This only reflects the practical mini-batch implementation of the same training objective. The model parameters *Θ* are updated using the Adam optimizer.

The proposed IGMLP differs from a conventional MLP in two essential aspects. First, it preserves the grouped structure of football event information through branch-wise encoding. Second, it introduces an information-guided gating mechanism to adaptively control the relative contribution of contextual, pressure-aware, and historical coordination information. This design is more consistent with the nature of football passing behavior and is therefore better suited to event-level football pass outcome prediction.
**Algorithm 1:** Training Procedure of the IGMLPInput: Training dataset $D={\left\{(x_{i},y_{i})\right\}}_{i=1}^{N},$ number of epochs *E*, mini-batch size B learning rate *η*, decision threshold τ.Output: Trained model parameters Θ.1.Initialize model parameters Θ.2.**for** *e* = 1 to *E* do3.    Shuffle D and divide it into mini-batches $\left\{B_{1},B_{2},\cdots \right\}$
4.    **for** each mini-batch B do5.     **for** each sample $(x_{i},y_{i})\in B$ do6.      Split the input into grouped feature vectors:$x_{i}=[x_{i}^{\left(c\right)},x_{i}^{\left(p\right)},x_{i}^{\left(h\right)}]$
7.      Compute branch-wise hidden representations for u∈{c,p,h}:
hi(u)=ReLU(W1(u)xi(u)+b1(u));zi(u)=ReLU(W2(u)hi(u)+b2(u))
8.      Concatenate branch representations: $z_{i}^{\left(cat\right)}=[z_{i}^{\left(c\right)};z_{i}^{\left(p\right)};z_{i}^{\left(h\right)}]$
9.      Compute gate logits: $a_{i}=W_{g}z_{i}^{\left(cat\right)}+b_{g}$
10.      Compute normalized gate weights for u∈{c,p,h}:
$g_{i}^{\left(u\right)}=\exp(a_{i}^{\left(u\right)})/\sum_{u\in \{c,p,h\}}\exp(a_{i}^{\left(u\right)})$
11.      Fuse grouped representations: $z_{i}=\sum_{u\in \{c,p,h\}}g_{i}^{\left(u\right)}z_{i}^{\left(u\right)}$
12.       Compute classifier output:       oi=ReLU(Wozi+bo);
$s_{i}=W_{s}o_{i}+b_{s};$
${\hat{p}}_{i}=\sigma (s_{i})$
13.      Predict the pass outcome: ${\hat{y}}_{i}=1,\;if\;{\hat{p}}_{i}\geq \tau ;\;{\hat{y}}_{i}=0,\;otherwise$
14.     
**end for**
15.     Compute the mini-batch binary cross-entropy loss:       $L(\Theta )=-\frac{1}{\left|B\right|}\sum_{(x_{i},y_{i})\in B}\left[y_{i}\log({\hat{p}}_{i})+(1-y_{i})\log(1-{\hat{p}}_{i})\right]$
16.     Update model parameters:       $\Theta \leftarrow \Theta -\eta \nabla_{\Theta }L(\Theta )$
17.    
**end for**
18. 
**end for**
19.return Θ.

## 4. Experimental Validation and Discussion

### 4.1. Experimental Setup

To evaluate the effectiveness of the proposed IGMLP, a series of experiments were conducted on the constructed event-level football pass dataset. The experimental validation was designed to examine whether the proposed information-guided grouped modeling strategy can improve pass outcome prediction compared with representative machine learning and neural network models.

After event extraction, feature construction, and data cleaning, a total of 95,747 valid pass samples were used for model training and evaluation. To reduce potential information leakage and better simulate realistic football analytics scenarios, the samples were sorted according to match time and then split chronologically into training, validation, and test subsets.

Specifically, the dataset was divided as follows:

(32) $$N_{train}=67022,\;N_{val}=14362,\;N_{test}=14363$$

where $N_{train}$, $N_{val}$, and $N_{test}$ denote the numbers of samples in the training, validation, and test sets, respectively. The training set was used to optimize model parameters, the validation set was used for threshold selection and model monitoring, and the test set was used only for final performance evaluation.

The main experiments were conducted under two settings. The first one is the prediction path, which uses contextual, pressure-aware, and historical coordination features. This setting represents the main task of this study because it relies only on pre-event or event-synchronous information. The second one is the recognition path, where receipt-related cues are additionally included as auxiliary post-event information. Although the recognition path is not the primary focus of this study, it was retained as an auxiliary comparison to examine the performance upper bound when richer event cues are available.

(1) Evaluation Metrics

Since football pass outcome prediction is a binary classification problem, five commonly used classification metrics were adopted: Accuracy, Precision, Recall, F1-score, and area under the receiver operating characteristic curve (AUC). These metrics are defined as follows:

(33) Accuracy=TP+TNTP+TN+FP+FNPrecision=TPTP+FPRecall=TPTP+FNF1=2×Precision×RecallPrecision+Recall

where *TP*, *TN*, *FP*, and *FN* denote the numbers of true positive, true negative, false positive, and false negative samples, respectively. AUC was used to evaluate the threshold-independent discriminative ability of each model.

Among these metrics, F1-score and AUC are particularly important in this study. F1-score reflects the balance between Precision and Recall, while AUC evaluates the overall ranking and discrimination ability of the model. Therefore, they are used as the main indicators for comparing the stability and predictive capability of different models.

(2) Baseline Models

To comprehensively validate the performance of the proposed IGMLP, five representative baseline models were selected for comparison, covering standard feedforward neural networks, residual neural structures, convolutional neural networks, sequence learning models, and ensemble tree-based classifiers. As summarized in Table 2, the standard MLP was used to examine whether the proposed grouped modeling strategy can outperform direct feature concatenation. In particular, the standard MLP can be regarded as a non-grouped and non-gated reference model, because it directly concatenates all input variables and does not use branch-wise feature encoding or adaptive group-level fusion. Therefore, the comparison between the standard MLP and IGMLP directly examines whether the proposed grouped representation and gating mechanism provide additional benefits beyond a conventional MLP architecture. The Residual NN was introduced to assess whether a deeper residual structure benefits event-level pass outcome prediction. The 1D-CNN and LSTM models were selected as mature deep learning baselines to evaluate the effectiveness of convolutional and sequence-based feature modeling, respectively. In addition, the Boosting Tree model was included as a strong non-neural machine learning baseline. Through these comparisons, the proposed IGMLP can be evaluated against different representative modeling paradigms under the same dataset split and evaluation protocol.

To alleviate class imbalance, random oversampling was applied only to the minority class in the training set. Specifically, unsuccessful pass samples were randomly replicated until the numbers of successful and unsuccessful samples in the training set were balanced. The validation and test sets were not oversampled and were kept unchanged to preserve the original data distribution for threshold selection and final evaluation.

The main hyperparameter settings used for the proposed model and baseline models are summarized in Table 3. For all neural-network-based models, the Adam optimizer was used with an initial learning rate of 1 × 10^−3^. Random oversampling was applied only to the training set to alleviate class imbalance, while the validation and test sets were kept unchanged. The validation set was used for model monitoring and decision-threshold selection. Specifically, the classification threshold was selected from 0.05 to 0.95 with a step size of 0.01 according to the best validation F1-score. No test-set information was used during hyperparameter setting or threshold selection.

All models were implemented in MATLAB 2021a. For neural network models, the Adam optimizer was used for parameter optimization. The mini-batch size was set to 512 for the proposed IGMLP, and the maximum number of training epochs was set to 30. The learning rate was initialized as 1 × 10^−3^. For CNN- and LSTM-based models, the maximum number of epochs was set to 25. The validation set was used to monitor the training process and select the decision threshold that achieved a better balance between Precision and Recall. For all neural network models, the input numerical features were standardized using the mean and standard deviation calculated from the training set. Categorical variables were transformed into one-hot representations. To alleviate the influence of class imbalance, oversampling was applied to the minority class in the training set. The same train-validation-test split was used for all models to ensure fair comparison. For the proposed IGMLP, the hidden dimension of the branch encoder was set to 64, and the latent embedding dimension of each information group was set to 32. The embedding dimension was fixed at 32 as a compact architectural setting to balance representation capacity and model complexity. This setting provides sufficient latent space for each semantic feature group while avoiding an excessive number of parameters, which is important for maintaining a lightweight model on structured event-level data. The same embedding dimension was used in both the prediction and recognition paths to ensure a controlled and consistent model configuration. The model separately encoded contextual, pressure-aware, and historical coordination features and then adaptively fused their latent representations through the information-guided gating mechanism. In the recognition path, an additional receipt-related branch was included only for auxiliary analysis. The final model performance was evaluated on the independent test set. To reduce the influence of random initialization and sampling randomness, each model was independently run 30 times under the same data split and hyperparameter settings. For each run, the model was trained from scratch, and the validation set was used for decision-threshold selection. The final test performance was recorded for each independent run, and the average value was used for model comparison. In addition, paired statistical tests were performed based on the 30-run results to examine whether the performance differences between IGMLP and the baseline models were statistically significant.

### 4.2. Experimental Results Analysis

**Figure 4:**
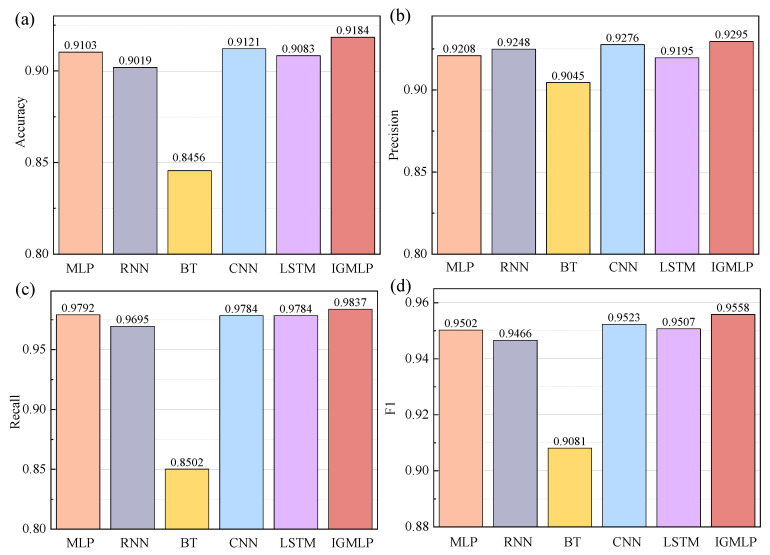
Performance comparison of different models on the prediction path. (**a**) Accuracy; (**b**) Precision; (**c**) Recall; (**d**) F1-score.

**Figure 5:**
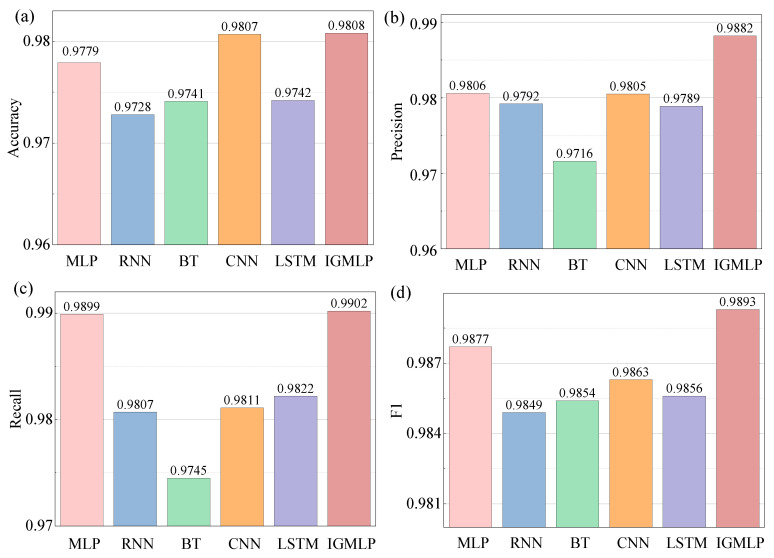
Performance comparison of different models on the recognition path. (**a**) Accuracy; (**b**) Precision; (**c**) Recall; (**d**) F1-score.

**Figure 6:**
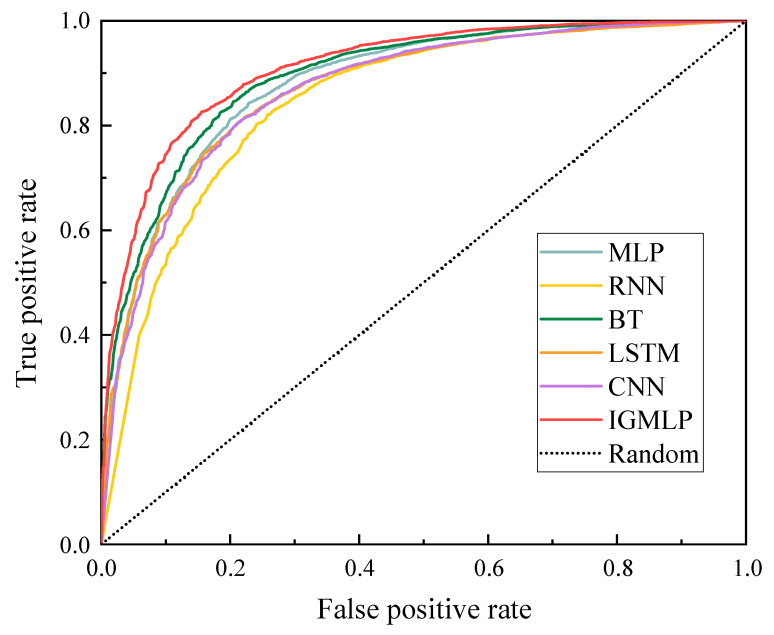
ROC curves of different models on the prediction path.

**Figure 7:**
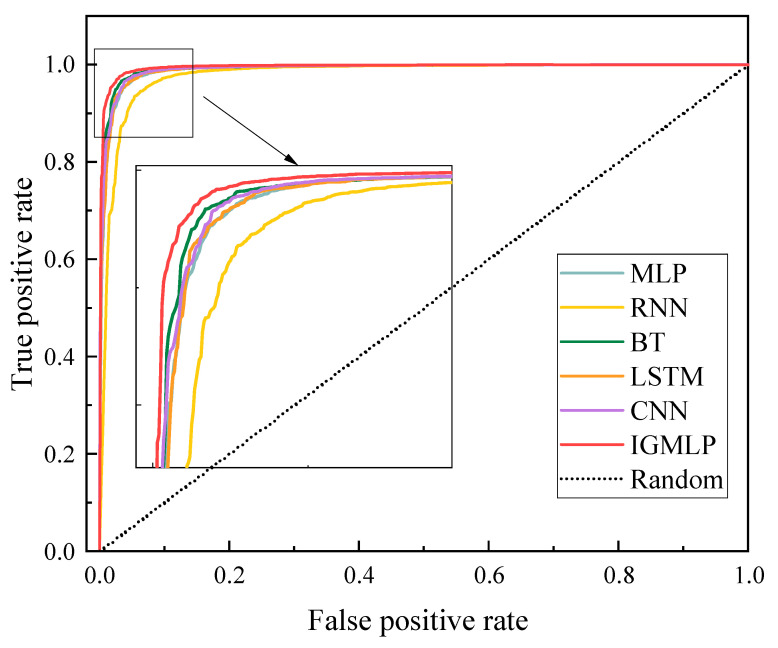
ROC curves of different models on the recognition path.

The experimental results are reported separately for the prediction and recognition paths. For the prediction path, Table 4 presents the average performance comparison of MLP, RNN, BT, CNN, LSTM, and the proposed IGMLP over 30 independent runs under the same data split and hyperparameter settings. Table 5 further reports the statistical comparison of accuracy, and Table 6 provides the model complexity comparison on the prediction path. For the recognition path, Table 7 presents the corresponding average performance comparison of different models over 30 independent runs. To provide a more intuitive comparison, Figure 4 and Figure 5 visualize the main evaluation indicators, including accuracy, precision, recall, and F1-score, using bar charts for the prediction and recognition paths, respectively. In addition, Figure 6 and Figure 7 show the ROC curves of different models on the prediction and recognition paths, respectively, further illustrating their discriminative ability under different classification thresholds. The proposed IGMLP achieves competitive or superior performance on both paths, indicating that the grouped feature representation and gated multilayer perceptron structure can effectively improve football pass outcome prediction.

For the prediction path, Table 4 shows that IGMLP obtains the best overall performance among all compared models. Specifically, IGMLP achieves an accuracy of 0.9184, a precision of 0.9295, a recall of 0.9837, an F1-score of 0.9558, and an AUC of 0.9325. Compared with the standard MLP, IGMLP improves the accuracy from 0.9103 to 0.9184, demonstrating the effectiveness of the grouped feature representation and gated fusion strategy. Although the numerical improvement in accuracy appears modest, the reported values are averaged over 30 independent runs. Therefore, the Wilcoxon signed-rank test was further performed to examine whether the improvement was statistically significant. As shown in Table 5, the improvement of IGMLP over the standard MLP is statistically significant, with *p* < 0.05. This indicates that the observed accuracy gain is not merely caused by random initialization or sampling fluctuations, but reflects a reliable performance improvement.

Compared with CNN and LSTM, IGMLP also achieves higher accuracy, recall, and F1-score, suggesting that the proposed model can better capture the nonlinear relationship between pass-context features and pass outcomes. Although BT obtains a relatively high AUC, its accuracy, recall, and F1-score are clearly lower than those of IGMLP, indicating that BT may have certain discriminative ability but performs less stably in terms of final classification results. The bar charts in Figure 4 further confirm these observations, where IGMLP consistently ranks at or near the top across the main evaluation indicators. In particular, the higher recall and F1-score indicate that IGMLP is more effective in identifying successful pass events while maintaining balanced classification performance.

To further evaluate the lightweight nature of the proposed framework, Table 6 compares the model complexity and inference efficiency of different models on the prediction path. Among the neural network models, IG-MLP contains only 13,764 trainable parameters, which is substantially fewer than those of MLP (18,242), LSTM (21,186), ResNet (84,290), and CNN (343,170). Although the boosting tree model does not have directly comparable trainable neural parameters, its inference efficiency is also reported for reference. In terms of inference time, IG-MLP required 203.173 s to complete prediction on the test set, which was lower than those of MLP, LSTM, CNN, and ResNet, while maintaining superior predictive performance. These results indicate that the proposed grouped feature representation and gated fusion strategy improve prediction effectiveness without introducing excessive computational burden, thereby providing a favorable trade-off between predictive accuracy and computational efficiency.

For the recognition path, Table 7 indicates that all models achieve relatively high performance, suggesting that the recognition task is more separable under the constructed feature representation. Different from the prediction path, the recognition path incorporates post-event receipt-related cues and is used as an auxiliary analysis to examine model behavior under richer event information. Therefore, the statistical comparison was mainly conducted for the prediction path, which represents the core pre-event pass outcome prediction task, while the recognition path is discussed through descriptive performance comparison. IGMLP achieves the highest accuracy of 0.9808 and the highest F1-score of 0.9893, showing its strong overall classification capability. CNN obtains a very close accuracy of 0.9807 and an F1-score of 0.9893, indicating that CNN also performs well in the recognition path. BT achieves the highest precision of 0.9966 and the highest AUC of 0.9951, but its recall is only 0.9745, which is lower than those of MLP, RNN, CNN, LSTM, and IGMLP. This means that BT is more conservative in predicting positive samples and may miss some successful pass events. In contrast, IGMLP maintains a better balance between precision and recall, with a precision of 0.9882 and a recall of 0.9902. Figure 5 visually supports this conclusion, as IGMLP shows consistently high values across different indicators. Therefore, although some baseline models perform well on individual metrics, IGMLP provides a more balanced and reliable recognition performance.

The ROC curves in Figure 6 further demonstrate the superiority of IGMLP on the prediction path. The ROC curve of IGMLP is generally closer to the upper-left corner than those of the other models, indicating a higher true positive rate under the same false positive rate. This result is consistent with the AUC values reported in Table 4, where IGMLP achieves the highest AUC of 0.9325. The improved ROC performance suggests that IGMLP has stronger threshold-independent discriminative ability and can better distinguish successful and unsuccessful pass events. Compared with RNN, CNN, and LSTM, the proposed model shows a more favorable trade-off between sensitivity and specificity, which is important for event-level football pass outcome prediction.

Figure 7 presents the ROC curves of different models on the recognition path. Since the recognition path yields generally higher classification performance, the ROC curves of most models are concentrated near the upper-left region. Nevertheless, IGMLP still maintains a strong discriminative capability and remains highly competitive with the best-performing models. The enlarged region in Figure 7 provides a clearer view of the differences among models in the high-performance area. Combined with the results in Table 7, IGMLP achieves excellent overall performance with high accuracy, recall, F1-score, and AUC, indicating that the proposed model remains stable and effective when applied to the recognition path.

In summary, the experimental results demonstrate that the proposed IGMLP model provides robust and reliable performance for event-level football pass outcome prediction. On the prediction path, IGMLP achieves the best performance across all five-evaluation metrics, especially in AUC and F1-score. On the recognition path, IGMLP also shows strong competitiveness and maintains a good balance between precision and recall. These results verify that integrating information-guided contextual features into a lightweight multilayer perceptron framework can effectively enhance the modeling of pass outcomes from structured football event data.

### 4.3. Discussion

The experimental results confirm the effectiveness of the proposed IGMLP model for event-level football pass outcome prediction. Compared with the baseline models, IGMLP achieves more balanced performance in terms of accuracy, precision, recall, F1-score, and AUC, especially on the prediction path. This suggests that the information-guided feature representation strategy can effectively describe the local passing context and improve the model’s ability to distinguish successful and unsuccessful passes. By integrating passing-context features such as spatial information, defensive pressure, historical coordination, and receiver-related cues, the proposed model captures important nonlinear relationships in structured event data while maintaining a lightweight model structure. Therefore, reliable pass outcome prediction can be achieved without relying on overly complex deep learning architectures, which is beneficial for practical deployment in football analysis and training scenarios.

The findings also provide useful implications for youth football education. Passing performance in youth football is not only related to technical execution, but also closely associated with tactical awareness, spatial perception, and decision-making ability. The proposed model can help coaches identify contextual factors that influence pass success, such as pass distance, pressure level, and receiver position. Based on these results, coaches can design more targeted training tasks to improve players’ passing choices, off-ball support, and ability to make decisions under defensive pressure. In addition, event-level pass outcome prediction can support more objective and contextualized player evaluation. Traditional indicators such as pass completion rate may overlook the difficulty and tactical value of each pass. In contrast, the proposed model considers the specific context of each passing event, which can help coaches distinguish between low-risk successful passes and high-value but difficult passing attempts. This is meaningful for guiding young players to develop both technical accuracy and creative tactical decision-making.

**Figure 8:**
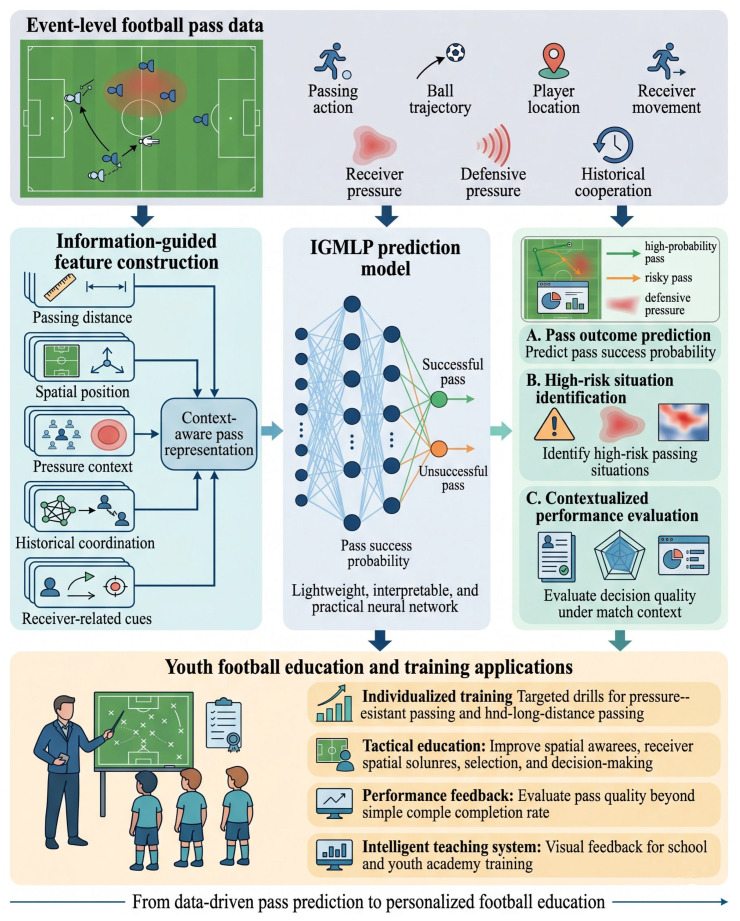
Key educational implications of event-level pass outcome prediction for youth football training.

To further summarize the educational implications of the proposed IGMLP model, the key concepts discussed in this section are visually organized in Figure 8. As shown in Figure 8, event-level pass outcome prediction can support youth football education from several aspects, including contextualized pass evaluation, tactical awareness development, decision-making training, pressure-resistant passing, and intelligent training feedback.

Nevertheless, this study still has some limitations. The current model mainly uses structured event-level features, while richer information from tracking data, video data, and physiological data has not yet been fully incorporated. Future work may combine multi-source football data and explainable artificial intelligence methods to further improve model interpretability and practical value in youth football training. In this sense, the proposed IGMLP model provides a useful data-driven tool for pass outcome prediction and offers potential support for intelligent football education and training.

Although the proposed IG-MLP achieved promising results on the constructed StatsBomb La Liga dataset, its generalization ability across other professional football leagues should be further examined. Different leagues may exhibit distinct tactical styles, pressing intensity, passing tempo, player movement patterns, and event annotation distributions, which may affect the learned feature representations and gate weights. Since the proposed framework is based on semantically defined feature groups rather than league-specific handcrafted rules, it has the potential to be transferred to other leagues after appropriate data preprocessing and feature alignment. Nevertheless, direct cross-league validation using data from other professional competitions, such as the English Premier League, Bundesliga, Serie A, or Ligue 1, remains an important direction for future work.

## 5. Conclusions

To address the problem that existing football prediction studies mainly focus on match-level outcomes while paying limited attention to fine-grained pass outcome prediction, this study proposed an information-guided multilayer perceptron model for event-level football pass outcome prediction. Based on structured football event data, multiple passing-context features were extracted, including spatial information, defensive pressure, historical coordination, and receiver-related cues. These features were then integrated into the proposed IGMLP framework to model the nonlinear relationship between in-game passing context and pass outcomes. The main conclusions are as follows:

(a) The proposed information-guided feature representation can effectively describe the local context of passing events. By considering not only basic passing attributes but also contextual and interaction-related information, the model provides a more comprehensive representation of the passing decision environment.
(b) The proposed IGMLP model achieves strong predictive performance on both the prediction and recognition paths. Compared with the baseline models, IGMLP shows more balanced performance in terms of accuracy, precision, recall, F1-score, and AUC, especially on the prediction path, demonstrating the effectiveness of the proposed method.
(c) The event-level pass outcome prediction framework provides useful support for football analysis and youth football education. The model can help identify key factors affecting pass success and provide data-driven evidence for contextualized player evaluation, tactical awareness training, and decision-making improvement.

Future work will further incorporate multi-source football data, such as tracking data, video information, and physiological indicators, to improve the richness of feature representation. In addition, explainable artificial intelligence methods will be introduced to better reveal the contribution of different contextual factors and enhance the practical value of the model in football training and intelligent sports education.

## Figures and Tables

**Table 1:** Distribution of successful and unsuccessful passes in the constructed dataset.

Subset	Total Samples	Successful Passes	Unsuccessful Passes	Success Rate
Training set	67,022	59,548	7474	88.85%
Validation set	14,362	12,914	1448	89.92%
Test set	14,363	12,891	1472	89.75%
Total	95,747	85,353	10,394	89.14%

**Table 2:** Baseline models used for comparison with the proposed IGMLP.

Model	Description
MLP	A standard multilayer perceptron using directly concatenated input features.
RNN	A residual neural network with skip connections, used to examine whether deeper nonlinear mapping improves prediction.
BT	A tree-based ensemble model, used as a strong non-neural machine learning baseline.
CNN	A one-dimensional convolutional neural network that treats the feature vector as a one-dimensional sequence.
LSTM	A long short-term memory network that models the feature vector in a sequence-like manner.
IGMLP	The proposed information-guided grouped MLP with branch-wise encoding and gated fusion.

**Table 3:** Hyperparameter settings of the proposed model and baseline models.

Model	Main Architecture/Learner Setting	Epochs/Cycles	Batch Size	Learning Rate	Other Settings
MLP	Fully connected layers with 128 and 64 hidden units	30	512	1 × 10^−3^	Dropout = 0.2; Adam optimizer; validation-based threshold selection
RNN	Projection layer with 128 hidden units and two residual blocks	30	512	1 × 10^−3^	Dropout = 0.2; Adam optimizer; validation-based threshold selection
BT	Logit Boost ensemble with decision-tree learners	300 cycles	—	0.05	MaxNumSplits = 20; MinLeafSize = 10; validation-based threshold selection
CNN	Three convolutional layers with 32, 64, and 128 filters	25	256	1 × 10^−3^	Kernel size = 3; batch normalization; dropout = 0.3; validation-based threshold selection
LSTM	LSTM layer with 64 hidden units followed by a 64-unit fully connected layer	25	256	1 × 10^−3^	Dropout = 0.2; Adam optimizer; validation-based threshold selection
IGMLP	Branch-wise encoders with group-level SoftMax gating	30	512	1 × 10^−3^	Branch hidden dimension = 64; embedding dimension = 32; weight decay = 1 × 10^−4^; validation-based threshold selection

**Table 4:** Average performance comparison on the prediction path over 30 independent runs.

Model	Accuracy	Precision	Recall	F1-Score	AUC
MLP	0.9103	0.9208	0.9792	0.9502	0.8819
RNN	0.9019	0.9248	0.9695	0.9466	0.8534
BT	0.8456	0.9045	0.8502	0.9081	0.9102
CNN	0.9121	0.9276	0.9784	0.9523	0.8737
LSTM	0.9083	0.9195	0.9784	0.9507	0.8698
IGMLP	0.9184	0.9295	0.9837	0.9558	0.9325

**Table 5:** Statistical comparison of accuracy on the prediction path.

Compared Models	Baseline Accuracy	IGMLP Accuracy	Accuracy Difference	*p*-Value
IGMLP vs. MLP	0.9103	0.9184	+0.0081	<0.05
IGMLP vs. RNN	0.9019	0.9184	+0.0165	<0.05
IGMLP vs. BT	0.8456	0.9184	+0.0728	<0.05
IGMLP vs. CNN	0.9121	0.9184	+0.0063	<0.05
IGMLP vs. LSTM	0.9083	0.9184	+0.0101	<0.05

**Table 6:** Model complexity comparison on the prediction path.

Model	Trainable Parameters	Inference Time (s)
MLP	18,242	231.602
RNN	84,290	498.751
BT	N/A	118.437
CNN	343,170	371.869
LSTM	21,186	286.536
IGMLP	13,764	203.173

**Table 7:** Average performance comparison on the recognition path over 30 independent runs.

Model	Accuracy	Precision	Recall	F1-Score	AUC
MLP	0.9779	0.9806	0.9899	0.9877	0.9903
RNN	0.9728	0.9792	0.9907	0.9849	0.9853
BT	0.9741	0.9966	0.9745	0.9854	0.9951
CNN	0.9807	0.9875	0.9911	0.9893	0.9914
LSTM	0.9742	0.9889	0.9822	0.9856	0.9891
IGMLP	0.9808	0.9882	0.9902	0.9893	0.9925

## Data Availability

All data generated and analyzed during this study are included in this published article.

## References

[1] Gaddour M., Nticha I., Mtawaa S., Dhahbi W., Chaabene H., Jemni S., Ben Saad H. (2026). Injury Prediction in Football: How Artificial Intelligence Is Shaping the Present and Transforming the Future in Africa. Sports Health.

[2] Elstak I., Salmon P., McLean S. (2024). Artificial intelligence applications in the football codes: A systematic review. J. Sports Sci..

[3] Martins F., Przednowek K., Santos F., França C., Martinho D., Gouveia É.R., Marques A., Sarmento H. (2025). Predictive models of injury risk in male professional football players: A systematic review. Inj. Prev..

[4] Brauers J.J., Den Hartigh R.J., Klooster D., Oosterveld F.G., Lemmink K.A., Brink M.S. (2026). The short-term relation between load and acute psychophysiological responses in football: A meta-analysis and methodological considerations. Sci. Med. Footb..

[5] Sun Y., Chu H. (2025). The outcome prediction method of football matches by the quantum neural network based on deep learning. Sci. Rep..

[6] Quan T., Luo Y. (2025). Advanced Football Match Winning Probability Prediction: A CNN-BiLSTM_Att Model with Player Compatibility and Dynamic Lineup Analysis. Int. J. Adv. Comput. Sci. Appl..

[7] Li Y., Hong Y. (2022). Prediction of football match results based on edge computing and machine learning technology. Int. J. Mob. Comput. Multimed. Commun..

[8] Lee J., Kim J., Kim H., Lee J.S. (2022). A Bayesian approach to predict football matches with changed home advantage in spectator-free matches after the COVID-19 break. Entropy.

[9] Stock E.V., da Silva R., Fernandes H.A. (2022). A physics-based algorithm to perform predictions in football leagues. Phys. A Stat. Mech. Its Appl..

[10] Alves R. (2025). SCORE: A convolutional approach for football event forecasting. Int. J. Forecast..

[11] Xie Q. (2025). Dynamic Tactical Image Recognition and Analysis in Football Matches Using Convolutional Neural Networks. Trait. Signal.

[12] Kausalya K., Sudha S. (2025). An optimized deep learning approach for detection and classification of player actions in football game. Entertain. Comput..

[13] Sun Y., Gu K. (2024). Prediction of football players’ value in the transfer market of well-known european leagues based on FIFA 19 and real-world data. Int. Arab J. Inf. Technol..

[14] Nengchao P. (2023). Research on physical fitness training of football players based on improved LSTM neural network to improve physical energy saving and health. 3C Tecnol..

[15] Liao S., Fu C. (2025). The optimization of youth football training using deep learning and artificial intelligence. Sci. Rep..

[16] Dijkhuis T.B., Kempe M., Lemmink K.A. (2021). Early prediction of physical performance in elite soccer matches—A machine learning approach to support substitutions. Entropy.

[17] Hassan B.M., Algarni F., Alshamrani R., Althbiti A., Albalawi A., Ismail A. (2026). Football cybersecurity threat severity prediction using multi-head transformer-based deep learning models. Sci. Rep..

[18] Empacher C., Kamps U., Volovskiy G. (2023). Statistical prediction of future sports records based on record values. Stats.

[19] Anzer G., Bauer P. (2022). Expected Passes: Determining the Difficulty of a Pass in Football (Soccer) Using Spatio-Temporal Data. Data Min. Knowl. Discov..

[20] Power P., Ruiz H., Wei X., Lucey P. (2017). Not All Passes Are Created Equal: Objectively Measuring the Risk and Reward of Passes in Soccer from Tracking Data. Proceedings of the 23rd ACM SIGKDD International Conference on Knowledge Discovery and Data Mining, Halifax, NS, Canada, 13–17 August 2017.

[21] Goes F.R., Meerhoff L.A., Bueno M.J.O., Rodrigues D.M., Moura F.A., Brink M.S., Elferink-Gemser M.T., Knobbe A.J., Cunha S.A., Lemmink K.A.P.M. (2022). A Risk-Reward Assessment of Passing Decisions: Comparison between Positional Roles Using Tracking Data from Professional Men’s Soccer. Sci. Med. Footb..

[22] Hassani K., Ramdani M., Lotfi M. (2025). Dynamic expected threat (DxT) model: Addressing the deficit of realism in football action evaluation. Appl. Sci..

[23] Fernandez J., Bornn L. Wide Open Spaces: A Statistical Technique for Measuring Space Creation in Professional Soccer. Proceedings of the MIT Sloan Sports Analytics Conference.

[24] Fernández J., Bornn L., Cervone D. (2021). A Framework for the Fine-Grained Evaluation of the Instantaneous Expected Value of Soccer Possessions. Mach. Learn..

[25] Decroos T., Bransen L., Van Haaren J., Davis J. (2019). Actions Speak Louder than Goals: Valuing Player Actions in Soccer. Proceedings of the 25th ACM SIGKDD International Conference on Knowledge Discovery and Data Mining, Anchorage, AK, USA, 4–8 August 2019.

[26] Midoglu C., Kjæreng Winther A., Boeker M., Dahl Pettersen S., Pedersen S., Ragab N., Kupka T., Hicks S.A., Randers M.B., Jain R. (2024). A large-scale multivariate soccer athlete health, performance, and position monitoring dataset. Sci. Data.

